# Legal Standards for Selenium Enriched Foods and Agricultural Products: Domestic and International Perspectives

**DOI:** 10.3390/nu16213659

**Published:** 2024-10-28

**Authors:** Xiao Ren, Yuchen Wang, Junmao Sun, Kehong Liang, Hong Zhu, Yanmi Li, Jieying Gao, Yimin Zhang, Shuxian Huang, Dazhou Zhu

**Affiliations:** Institute of Food and Nutrition Development, Ministry of Agriculture and Rural Affairs, Beijing 100081, China; rxiao1022@163.com (X.R.); 82101232184@caas.cn (Y.W.); liangkehong@caas.cn (K.L.); zhuhong@caas.cn (H.Z.); liyanmi@stu.cdu.edu.cn (Y.L.); gaojieying203@126.com (J.G.); zz8978681@163.com (Y.Z.); 821012450674@caas.cn (S.H.)

**Keywords:** selenium enrichment, legal standards, foods, agricultural products

## Abstract

**Background/Objectives:** Selenium is indispensable for human health, yet vast regions worldwide grapple with selenium-deficient soils, rendering dietary intake a critical avenue for supplementation. This narrative review aims to systematically examine and compare domestic and international regulations and standards related to selenium enrichment, providing insights to enhance regulatory frameworks and standardization within the selenium-enrichment industry. **Methods:** From June to September 2024, we conducted a comprehensive search of official websites belonging to international organizations (e.g., Codex Alimentarius Commission, European Union) and governmental agencies of countries such as China and the United States. Keywords, like “selenium enrichment”, “selenium standards”, and “selenium detection methods”, were employed to identify pertinent regulations, standards, and guidelines encompassing intake benchmarks, detection methodologies, product specifications, technical guidelines for production, labeling requirements, and certification management norms. **Results:** Our analysis reveals several challenges within the current selenium-enriched regulatory and standardization systems, including inconsistent product determination criteria and limit settings, incomplete technical guidelines for selenium-enriched agricultural production, and a lack of unified regulations for labeling selenium-enriched agricultural products. **Conclusions:** These findings underscore the need for harmonization of standards and enhanced regulatory oversight. To address these issues, we recommend bolstering safety risk assessments for selenium-enriched agricultural products, establishing and refining a comprehensive standard system for selenium-enriched agriculture, and intensifying quality and safety supervision. This study offers a valuable reference for policymakers and stakeholders to promote the sustainable development of the selenium-enrichment industry.

## 1. Introduction

**Figure 1:**
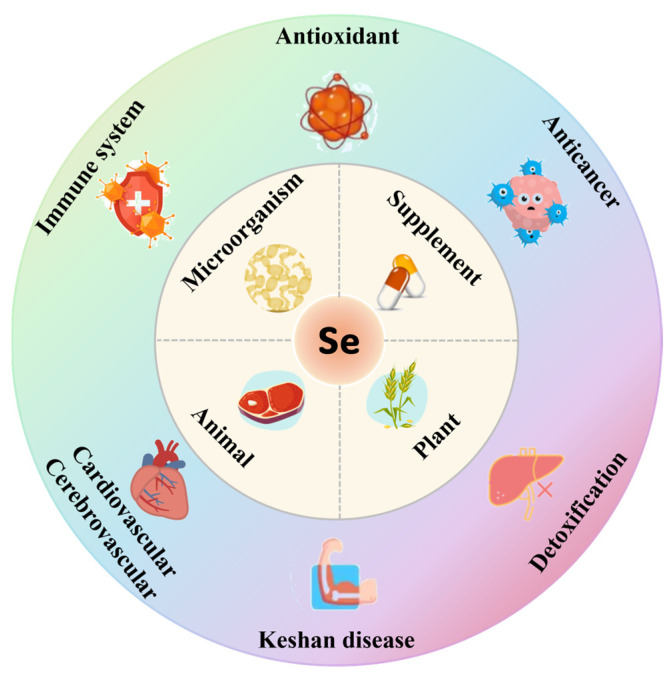
Source and function of selenium.

Selenium is an indispensable trace element, crucial for the physiological activities of humans and animals. Since the groundbreaking discovery by Schwarz and Foltz in 1957 that selenium can prevent liver necrosis in rats [1], it has been progressively acknowledged as an essential micronutrient. This pivotal discovery was the first to illuminate selenium’s essential physiological role in living organisms, leading the scientific community to acknowledge that selenium is not only critical for animal health but may also play an equally significant role in human nutrition. Consequently, research on selenium expanded into the domain of human health, spurring further exploration of its physiological functions and nutritional importance. To date, 25 seleno-proteins have been identified in the human proteome, all of which perform their functions through selenocysteine residues. Prominent seleno-proteins include glutathione peroxidase, thioredoxin reductase, and iodothyronine deiodinase [2]. Extensive research has demonstrated that selenium exhibits antioxidant properties [2,3,4], possesses anti-cancer potential [5,6], supports immune system functions [7,8], facilitates detoxification [9,10], and helps prevent cardiovascular diseases [11,12]. Selenium deficiency can lead to ailments such as Keshan disease and Kashin–Beck disease [13,14] (Figure 1).

Selenium naturally occurs in soil, water, and air; however, its global distribution within soils is markedly uneven. Regions exhibiting high soil selenium concentrations include parts of Wyoming and the Dakotas in the United States [15], Enshi in China, and specific areas of Ireland, Brazil, Colombia, and Venezuela [16]. Conversely, certain regions—such as Finland (prior to the addition of selenium to fertilizers in 1984) [17], central Serbia, the belt stretching from northeastern to central–southern China, the Congo, New Zealand, Ukraine, Germany, the United States, and Oceania—are significantly selenium-deficient. Notably, Australia encompasses both high-selenium and low-selenium soils [18].

China is recognized as a selenium-deficient country, with approximately 51% of its soils lacking this essential element [19]. In most regions, the concentrations of selenium in soil, water, plants, human diets, and even human hair are notably low. A survey by the Ministry of Natural Resources revealed that China’s naturally selenium-enriched land covers only about 121 million mu (8.07 million hectares), leaving 70% of the nation deficient in selenium. Low selenium levels are prevalent in Tibet, southern Xinjiang, and parts of Inner Mongolia, Shanxi, and Qinghai. In contrast, elevated selenium concentrations are found in the southwestern provinces—Hunan, Guizhou, Guangxi, Guangdong, and Chongqing—where they are widely distributed in large-scale epithermal mineralization zones and Lower Cambrian black shales, with scattered occurrences in Xinjiang, Gansu, Sichuan, and Yunnan [20].

**Figure 2:**
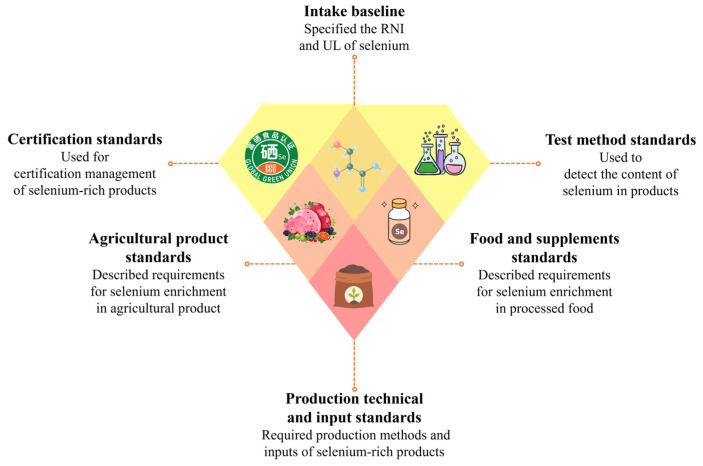
Standard types related to selenium.

Selenium is an indispensable trace element for the human body, conferring numerous health benefits, such as antioxidant effects, immune system enhancement, and the prevention of cardiovascular diseases. However, the safe intake range of selenium is relatively narrow; insufficient intake can lead to health complications, while excessive consumption may cause selenium toxicity [21]. Therefore, maintaining an appropriate selenium intake is crucial for health. In many regions worldwide, geological conditions or dietary patterns result in inadequate selenium levels in residents’ diets, necessitating supplementation through dietary supplements or selenium-enriched foods. Against this backdrop, the selenium-enrichment industry has developed rapidly, becoming a critical means of addressing selenium deficiency. As an early pioneer in research on selenium and its health impacts, China has provided invaluable data references for other nations. To better regulate the high-quality advancement of this industry and enhance the scientific foundation of its products, many countries have established relevant standards and regulations. These include intake reference values, selenium detection methodologies, product specifications, production technical guidelines, labeling requirements, and certification management norms, among others. However, several issues persist within the current standards for selenium-enrichment. It is imperative to establish a more comprehensive standard system encompassing the entire industrial chain to provide scientific and theoretical support for the regulation and development of the selenium-enrichment industry (Figure 2).

## 2. Methods

This narrative review aims to systematically synthesize domestic and international regulations and standards related to selenium enrichment, compare the regulatory approaches of different countries and organizations in this field, identify common challenges, and offer recommendations for future regulatory enhancements. Conducted between June and September 2024, the study primarily accessed official websites of international organizations and national governments, professional information platforms, and academic databases to collect relevant regulations, standards, and literature.

Initially, we explored the official websites of international organizations, such as the European Food Safety Authority (EFSA), the Codex Alimentarius Commission (CAC), and the International Organization for Standardization (ISO), to obtain globally recognized regulations and standards on selenium enrichment. We also reviewed official national government websites, including China’s National Health Commission, the State Administration for Market Regulation, the Ministry of Agriculture and Rural Affairs, the U.S. Food and Drug Administration (FDA), the National Academy of Medicine (NAM), the Code of Federal Regulations (CFR), and the Official Journal of the European Union, to gather the latest regulations and standards from various countries. Additionally, we utilized Foodmate to collect summarized information on Chinese food standards and regulations. Although Foodmate is not an official government website, the standards it provides are in the form of official documents, ensuring their authority and accuracy.

Using these websites and platforms, we conducted keyword searches, such as “selenium enrichment”, “selenium standards”, “selenium testing methods”, “food safety standards”, “selenium intake”, “selenium nutritional supplements”, and “selenium-enriched agricultural products”. The search was confined to the Chinese and English languages, focusing on regulations and standards published up to September 2024. We initially reviewed titles and abstracts to preliminarily select regulations, standards, and literature closely related to the research topic. For documents meeting the inclusion criteria, we obtained the full texts and extracted the necessary information.

The inclusion criteria encompassed valid regulations, standards, and guidelines officially published by government departments or international organizations, covering topics such as selenium safety assessments, intake reference values, testing methods, quality standards for selenium-enriched foods and supplements, nutritional supplement usage standards, labeling requirements, selenium-enriched agricultural production protocols, and certification management standards. Excluded were outdated versions of documents, unofficial drafts, internal discussion papers, unpublished consultation documents, and literature unrelated to the research topic.

From the included documents and literature, we extracted basic information, such as titles, standard numbers, issuing agencies, publication dates, and implementation dates, along with key content, including recommended nutrient intake (RNI), tolerable upper intake levels (UL), testing method standards, quality standards for selenium-enriched foods and supplements, usage standards for nutritional supplements, labeling requirements, selenium-enriched agricultural production protocols, and certification management standards. The extracted information was categorized and organized by country or region and by topic, employing comparative analysis to identify similarities, differences, and common challenges in selenium enrichment regulation across countries.

To ensure the accuracy and reliability of the information, all collected regulations, standards, and literature were sourced directly from official websites, authoritative information platforms, or reputable academic databases. We verified the publication dates and validity of each document to avoid citing outdated or obsolete versions. For key data, cross-verification was performed using multiple sources to ensure completeness and consistency.

However, this study has geographical and linguistic limitations. Due to language and access constraints, regulations and standards published in languages other than Chinese or English were not fully collected, potentially omitting relevant information from some countries or regions. Additionally, regulations and standards may have been updated after the completion of this research, and this study does not cover changes beyond August 2024.

As a narrative review, this study aims to comprehensively compile and analyze existing regulations, standards, and literature to provide scientific evidence and policy recommendations for the advancement of the selenium enrichment industry.

## 3. Standards for Selenium Safety Assessment and Intake Reference

Both selenium deficiency and excess occur naturally worldwide, each imparting adverse health effects. Selenium deficiency can lead to diseases such as Keshan disease and Kashin–Beck disease, while excessive intake poses a risk of toxicity [2]. Research has demonstrated that excessive selenium intake can result in health issues, including hair loss, nail shedding, skin lesions, neurological disorders, and an increased risk of conditions like diabetes [22]; in severe cases, it may even lead to paralysis and death [23]. In the 1960s and 1970s, regions such as Enshi in Hubei Province, China, experienced incidents of acute selenium poisoning among local populations. Residents exhibited symptoms including hair loss, nail detachment, scalp itching and pain, and skin rashes. Investigations revealed that these health issues were caused by excessive selenium intake, with daily consumption exceeding 3000 μg—far surpassing the safe upper limit. This overconsumption was attributed to the high selenium content in the local environment. Selenium-rich stone coal, containing up to 80,000 mg/kg of selenium, was extensively used in daily life and agricultural activities [24]. Villagers applied coal ash, rich in soluble selenium, to their farmland, leading to excessive selenium accumulation in crops, such as corn. Additionally, residents often burned stone coal indoors to dry grain, resulting in selenium contamination of food due to coal smoke. These factors facilitated the entry of selenium into the human body through the food chain, causing poisoning. Vinceti et al. [25] conducted a systematic review and meta-analysis, revealing a significant positive correlation between high selenium exposure and an elevated risk of diabetes. Non-experimental studies showed a linear increase in diabetes risk among individuals with higher plasma or serum selenium levels. Experimental studies indicated that participants supplementing with 200 micrograms of selenium per day had an 11% higher risk of developing diabetes compared to the placebo group [26]. Thompson et al. [27] conducted a randomized, placebo-controlled trial to investigate the effects of selenium supplementation on the prevention of colorectal adenomas and the risk of type 2 diabetes. The study concluded that, while selenium may have some effect in reducing the risk of adenoma recurrence in patients with advanced adenomas, it also increased the risk of type 2 diabetes in older adults. Therefore, selenium supplementation is not recommended for populations with adequate selenium levels. Demircan et al. examined the complex, gender-specific relationship between selenium and type 2 diabetes through secondary analyses and observational studies. They concluded that selenium intake may increase the risk of diabetes in men, whereas appropriate supplementation in selenium-deficient women may help reduce this risk [28].

The global variation in soil selenium content is significant, leading economically developed countries to establish different selenium intake guidelines based on their dietary habits, environmental factors, and health considerations. To compare these standards, we have compiled the recommended intake levels and tolerable upper intake levels (ULs) from countries such as the United States, the European Union, Australia, and Japan (Table 1). Despite differences in soil selenium content, these countries have developed relatively mature standard systems, providing valuable insights into global selenium nutrition. This study focuses on universally applicable nutritional standards and, therefore, regions with abnormally high selenium content are not included in the table.

An international consensus has been established on the safe intake levels of selenium, culminating in the formulation of baseline standards, such as recommended daily allowances and upper safe limits. Countries and organizations—including China (1988), the World Health Organization (WHO) (2001), the United States (2000), Japan (2005), Canada (2010), and the European Union (2011)—have successively promulgated these standards for various demographic groups, including infants, children, adult males, adult females, pregnant women, and breastfeeding mothers [29].

**Figure 3:**
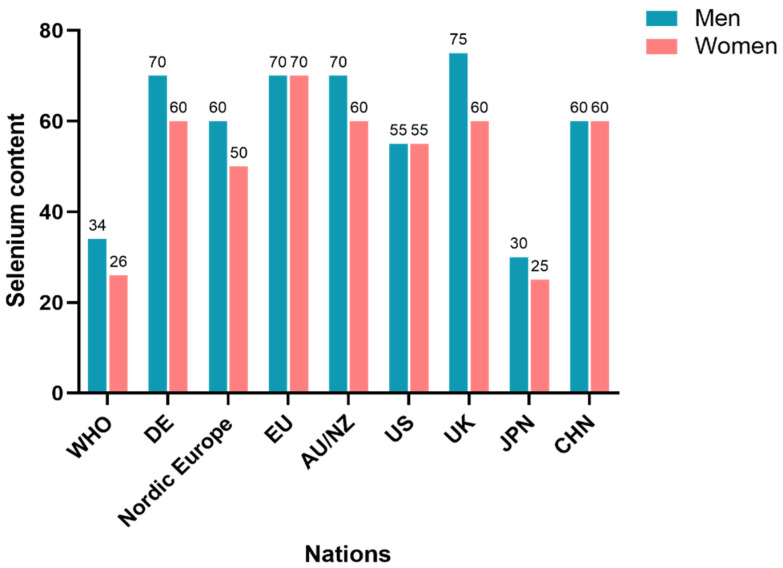
Recommended selenium intake by country.

Globally, the recommended selenium intake for humans ranges from 6 to 75 μg/day, increasing progressively with age. Adults require more than four times the intake of infants, and the UL is 5 to 10 times the recommended amount. Internationally, Japan has the lowest RNI for adult males (30 μg/day), the United Kingdom the highest (75 μg/day), while the United States and China fall in between (55 μg/day and 60 μg/day, respectively). The UL for adults is generally established at 400 μg/day (Table 1, Figure 3).

### 3.1. China

During the 1960s, incidents of acute selenium poisoning were reported in high-selenium regions of China, where individuals experienced complete hair loss after consuming selenium-rich corn, with intake levels reaching up to 38 mg/day. Moreover, numerous cases of chronic selenium poisoning were documented in these areas, manifesting primarily as hair loss and nail deformities [30]. Between 1982 and 1992, the Chinese Academy of Preventive Medicine conducted studies on the toxic and side effects of selenium in humans residing in high-selenium regions, such as Enshi. This pioneering research was the first to suggest that a daily intake exceeding 800 μg of selenium could induce toxic side effects. In 1988, taking safety factors into account, the Chinese Nutrition Society proposed recommendations for the daily RNI and UL of selenium for different population groups in China [31]. In 2017, China established the daily RNI of selenium for adults at 60 μg and the UL at 400 μg. For infants and children, the recommended daily intake decreases with age, and intakes exceeding 80 μg may elevate the risk of type 2 diabetes. The lowest observed adverse effect level (LOAEL) and the no observed adverse effect level (NOAEL) for adults are 900 μg/day and 600 μg/day, respectively. Using the NOAEL as a reference, the tolerable upper intake level (UL) is calculated by applying an uncertainty factor (UF) of 1.5, yielding UL = 600 μg/day ÷ 1.5 = 400 μg/day [32] (Table 1).

### 3.2. Other Countries and International Organizations

The WHO prescribes that the recommended selenium intake varies by age and gender: for infants and young children, ranging from 6 to 21 μg/day; for adolescents, it is set at 34 μg/day; for adult women, 26 μg/day; and for adult men, 34 μg/day. The UL for adults is established at 400 μg/day (Table 1).

The European Food Safety Authority (EFSA) Panel on Nutrition, Novel Foods and Food Allergens (NDA) conducted a systematic literature review to identify the clinical impacts of excessive selenium intake, potential biomarkers of effect, risks of chronic diseases, and evidence of neuropsychological developmental disorders in humans. Hair loss, an early observable manifestation of selenium overexposure and a well-established adverse effect, was chosen as the critical endpoint for determining selenium’s UL. In a large randomized controlled trial, a daily intake of 330 μg was identified as the LOAEL, upon which an uncertainty factor of 1.3 was applied. Consequently, the UL for adult males and females, including pregnant and lactating women, was established at 255 μg/day. For children, the UL was derived from the adult UL, adjusted according to body weight raised to the power of 0.75. Based on current intake data, the average adult is unlikely to exceed the UL unless regularly consuming high-dose selenium supplements or large quantities of selenium-enriched foods. Current selenium intake levels from food sources (excluding supplements) in European countries do not indicate any risks for infants and children, and intake from naturally occurring selenium in foods does not pose safety concerns. For infants and children, the use of selenium supplements should be approached cautiously and tailored to individual needs [33]. The stability of plasma seleno-protein concentrations indicates that selenium is adequately supplied to all tissues and reflects the saturation of functional selenium, thereby ensuring that selenium requirements are met. Accordingly, the European Union has set an adequate intake (AI) for adults at 70 μg/day (Table 1).

A study conducted in the United States involving 142 farm workers from Wyoming and South Dakota revealed that, even when selenium intake reached as high as 724 μg/day, the participants did not exhibit evident symptoms of toxicity. To safeguard sensitive individuals, the UF employed in establishing the UL in the United States is set at 2. Consequently, the UL was determined to be 400 μg/day. This standard applies to all adults, including pregnant and lactating women. For infants and children, the UL is calculated based on body weight, incrementally increasing from 45 μg/day for ages 0–6 months to 400 μg/day for ages 14–18 years. The recommended nutrient intake (RNI) for selenium is predicated on the amount required to maximize the synthesis of the seleno-protein glutathione peroxidase, assessed by the plateau in the enzyme’s plasma isoform activity. It is established at 55 μg/day for both men and women [34] (Table 1).

Australia and New Zealand have established the UL for adult selenium at 400 μg/day. For infants and young children, the UL is set at 45 μg/day, based on studies examining selenium content in human milk where no toxic reactions were observed within this intake range. The UL for children and adolescents is extrapolated from infant data and calculated according to body weight [35].

The estimated average requirement (EAR) for adults is grounded in experimental data from Duffield et al. [36] and Xia et al. [37], which assessed the impact of varying selenium supplementation levels on plasma seleno-protein concentrations. These results were adjusted for a reference adult body weight. The RDI assumes a coefficient of variation of 10% for the EAR. Both the EAR and RDI are rounded to the nearest 5 μg in the final data presentation; however, the unrounded EAR is used to estimate the RDI before rounding [35] (Table 1).

According to reports on chronic selenium poisoning in China, the NOAEL for selenium is estimated to be 13.3 μg/day. However, epidemiological studies have found that long-term supplementation of 200 μg/day in selenium-sufficient individuals increases the incidence of type 2 diabetes. This suggests that supplementing selenium at this level can have adverse effects when adequate selenium is already obtained from other sources. In Japan, the average selenium intake is estimated to be around 100 μg/day, which surpasses the recommended dietary allowance (RDA) for selenium. Consequently, the lowest observed adverse effect level for males aged 30–49 is set at 300 μg/day, as their average body weight (68.5 kg) is the highest among all gender and age groups. For other genders and age groups—including children and adolescents—the lowest intake was extrapolated to 300 μg/day based on body weight [38] (Table 1).

## 4. Standards for Selenium Detection Method

The margin between the recommended daily intake and the UL of selenium is relatively narrow, and selenium exists in two primary forms: organic and inorganic. Organic selenium is less toxic and is more readily absorbed and utilized by the human body [39]. Data indicate that the lethal dose (LD_50_) of seleno-methionine ranges from 4 to 26 mg/kg, while that of Se-methyl-selenocysteine is between 9 and 13 mg/kg. The minimum lethal dose for the inorganic form of selenium, sodium selenite, varies from 7 to 16 mg/kg [40].

Currently, the principal methods for determining selenium content in foods and beverages include Inductively Coupled Plasma Mass Spectrometry (ICP–MS), Atomic Absorption Spectroscopy (AAS), and High-Performance Liquid Chromatography coupled with Mass Spectrometry (HPLC–ICP–MS) [41]. ICP–MS is renowned for its high sensitivity and precision, capable of detecting trace concentrations of selenium and analyzing its various species. However, the equipment is costly, and its operation is complex. AAS, available in variants such as Flame AAS (FAAS), Graphite Furnace AAS (GF-AAS), and Hydride Generation AAS (HG-AAS), is suitable for quantifying total selenium at a lower cost, though it cannot provide information on selenium speciation. HPLC–ICP–MS enables the separation and detection of different selenium species, such as seleno-proteins and seleno-amino acids, but requires complex sample preparation and is associated with high costs. Each of these methods has distinct potential applications in the analysis of functional foods; however, their practical implementation necessitates careful consideration of factors such as sample type, desired analytical outcomes, equipment cost, and laboratory resources. Additionally, issues like instrumental interferences and matrix effects must be managed. For example, when detecting high concentrations of metal impurities, ICP–MS may need to be coupled with a Dynamic Reaction Cell (DRC) to enhance detection accuracy. Various countries have established selenium detection standards to quantify selenium content in selenium-enriched products. Notably, China has not only set standards for total selenium determination but has also developed protocols for measuring different chemical forms of selenium in food.

### 4.1. China

China’s standards for measuring selenium content encompass national standards, agricultural industry standards, and import–export industry standards, supplemented by various local and group standards. These regulations address the determination of total selenium, inorganic selenium, and organic selenium in a variety of products, including foodstuffs, edible mushroom powder, grains and grain products, livestock and poultry meat, seleno-proteins, cereals, and export commodities. The primary analytical methods employed are atomic fluorescence spectrometry, fluorescence spectrophotometry, and inductively coupled plasma mass spectrometry (ICP–MS) (Table 2).

### 4.2. Other Countries and International Organizations

The International Organization for Standardization (ISO) has issued standards for measuring selenium, focusing primarily on water quality and infant formula. The European Union has established protocols for determining selenium in foodstuffs using hydride generation atomic absorption spectrometry. In the United States, a range of methodological standards has been developed to detect selenium and its compounds in infant formula and other food products (Table 3).

## 5. Standards for Selenium-Enriched Foods and Supplements

Dietary intake remains a vital source of selenium supplementation for populations, and the development of selenium-enriched foods can more effectively meet this demand [57]. While dietary intake remains the primary source of selenium for most populations, different geographic regions face unique challenges in achieving adequate selenium levels. In selenium-deficient areas—such as parts of Europe, New Zealand, and regions of northeastern and southwestern China—low soil selenium content leads to insufficient selenium in crops and livestock, resulting in inadequate dietary intake among residents. This deficiency can potentially cause health problems associated with selenium scarcity. Conversely, in selenium-rich areas, like certain parts of the United States and Enshi in China, high soil selenium concentrations lead to excessive selenium content in food, increasing the risk of overconsumption. Moreover, factors such as climate change, agricultural practices, and dietary habits influence the selenium content of foods and overall intake. For instance, highly processed foods may result in selenium loss, while vegetarians may be at risk of inadequate selenium intake due to their dietary patterns. Therefore, it is crucial to adopt region-specific measures to optimize selenium intake. In selenium-deficient areas, strategies such as soil selenium supplementation, crop biofortification, and dietary supplements can help elevate selenium levels in the population. In selenium-rich regions, monitoring food selenium content and guiding residents toward balanced diets can help prevent overconsumption.

Selenium is incorporated into various multivitamin and mineral supplements, products containing vitamin E and other ingredients, as well as standalone formulations. Common forms include seleno-methionine, selenium-enriched yeast (cultivated in high-selenium media, primarily containing seleno-methionine), sodium selenite, and sodium selenate [58]. Naturally, selenium exists in two main forms: inorganic and organic. Inorganic selenium predominantly occurs as selenates and selenites, while organic selenium is mainly found as seleno-methionine and selenocysteine [59]. Supplements typically feature selenite (Se^4+^) or selenate (Se^6+^), seleno-methionine, Se-methyl-selenocysteine, and selenium-enriched yeast [60]. In response to the proliferation of selenium-enriched foods, countries have established mandatory regulations and standards. These standards focus primarily on two aspects: first, selenium-based health products; and second, the addition of selenium fortificants in food processing.

### 5.1. China

China has established a stringent approval and management system for selenium-based health products. The State Administration for Market Regulation (SAMR) is responsible for developing the list of permissible selenium sources in health products, as well as the technical specifications for their safe production, sale, and use. When companies develop selenium-based health products with specific health functions, they are required to submit them for registration or filing with SAMR, which then issues a National Health Product certificate and an approval number [61,62,63]. The standards for selenium fortifiers are formulated by health authorities. The 2011 National Food Safety Standard General Rules for Nutrition Labeling of Prepackaged Foods [64] stipulate that, when the nutrient reference value (NRV) for minerals in every 100 g of prepackaged food is ≥30%, the product can be labeled as “high” or “rich in”, meaning that when the selenium content reaches 18 μg per 100 g, it can be labeled as selenium enriched. The 2012 Standards for the Use of Nutrient Fortifiers [65] specify the allowable use of selenium in seven categories of food—formulated milk powder, rice, wheat flour, grain flour, bread, biscuits, and beverages—as well as the proportions of different selenium forms permitted. Additionally, quality standards for six selenium nutrient fortifiers, including selenium-enriched yeast, were subsequently issued (Table 4).

### 5.2. Other Countries and International Organizations

European and North American countries have established stringent approval systems for selenium-based health products authorized for production and sale, clearly defining product quality specifications, target populations, dosages, and contraindications. Major nations worldwide have developed comprehensive standards for adding selenium to common foods such as infant formula, bread, biscuits, and beverages.

The Codex Alimentarius Commission (CAC) has proposed guideline standards for labeling selenium-enriched foods. In the Codex Guidelines for Use of Nutrition and Health Claims (CAC/GL 23-1997) [74], the CAC specifies that, for nutrient claims related to vitamins and minerals—such as selenium—the nutrient content in the food must meet a specific proportion of the NRV to make such claims. Specifically, if the content of vitamins or minerals in a food product reaches 15% of the NRV, it can be claimed as a “source” of that nutrient. If the nutrient content is double the “source” standard, reaching 30% of the NRV, it can be claimed as “high in” that nutrient. For selenium, this means that the content should reach 16.5 μg per 100 g of food.

According to the CAC guidelines, the annexes of EU regulations 90/496/EEC [75] and (EU) No 1924/2006 [76] stipulate that the selenium content in food must reach 16.5 μg per 100 g to make such nutrient claims. The U.S. Food and Drug Administration (FDA) nutrition labeling rules state, that for a food to claim it is “rich in” a particular mineral—such as calcium, iron, or zinc—the content of that mineral in the food must be at least 20% or more of the daily value. In the United States, the recommended daily intake or DV for selenium is 55 μg. Therefore, to claim to be “rich in selenium”, a food must contain at least 11 μg per 100 g of selenium, which is 20% of 55 μg. In Australia and New Zealand, the threshold for labeling a food as selenium-enriched is set at 17.5 μg per 100 g (Table 5).

Regarding selenium supplements, under European Union regulations (EU) 2015/2283 [79] and (EU) 2023/938 [80], the yeast biomass of *Yarrowia lipolytica* has been approved as a novel food for use in dietary supplements. This yeast is suitable for adults and children over three years of age, with specific dosages varying by population subgroup. While the EFSA has evaluated the safety of *Yarrowia lipolytica*, no specific daily selenium intake standards have been established for it.

In the United States, the Food and Drug Administration (FDA), under the Dietary Supplement Health and Education Act (DSHEA), has issued a series of guidelines and regulatory documents for dietary supplements. These encompass Current Good Manufacturing Practices (CGMPs), regulations on new dietary ingredients, warning letters and safety information, labeling and claims requirements, adverse event reporting and record-keeping, and general compliance and inspection information for the industry. These guidelines extend to selenium dietary supplements.

Similarly, the Australia New Zealand Food Standards Code includes pertinent regulations [57]. These stipulate that selenium can be used as a nutrient fortifier in certain foods, in forms such as sodium selenite and seleno-methionine. The specific allowable amounts vary depending on the food type, with maximum permissible quantities usually specified in the reference intake for each category. For instance, in some cases, the maximum allowable addition of selenium is 25 μg per 100 g of food [81].

## 6. Standards for Selenium-Enriched Agricultural Products

Beyond processed foods, selenium-enriched agricultural products serve as a vital source of dietary selenium. Depending on their enrichment method, these products are categorized as either naturally selenium-enriched or artificially selenium-enriched. Such agricultural commodities predominantly originate from plants (e.g., tea, rice, garlic), animals (e.g., meat, eggs, dairy products), and microorganisms (e.g., yeast and fungi).

### 6.1. China

In addition to processed foods, selenium-enriched agricultural products serve as a significant source of dietary selenium. Depending on the method of enrichment, these products are categorized as naturally selenium-enriched or artificially selenium-enriched. Such agricultural commodities mainly originate from plants (e.g., tea, rice, garlic), animals (e.g., meat, eggs, dairy products), and microorganisms (e.g., yeast, fungi).

China has established recommended standards for selenium content in these agricultural products, along with supporting production guidelines. In 2008, a recommended national standard for selenium-enriched rice was developed. Since 2002, the Ministry of Agriculture and Rural Affairs has issued three recommended agricultural industry standards for selenium-enriched tea, potatoes, and garlic. Beginning in 2014, the All-China Federation of Supply and Marketing Cooperatives formulated three recommended standards covering selenium-enriched tea, a range of selenium-enriched agricultural products—including grains, beans, potatoes, vegetables, edible mushrooms, meat, eggs, and tea—and selenium-enriched potatoes.

Moreover, local governments in 12 provinces have issued 94 related local standards. Various industry associations and academic societies have released 181 group standards, primarily addressing selenium content in selenium-enriched agricultural products and associated production guidelines. Analysis of these standards reveals inconsistencies in the criteria and in the upper and lower limit settings for determining selenium-enriched agricultural products. All existing standards are recommended rather than mandatory, and the recommended selenium content indicators vary. Currently, there is no unified mandatory standard for defining selenium enrichment in primary agricultural products, nor are there regulations governing the labeling of selenium-enriched products. This regulatory gap has led to widespread issues of false advertising and consumer misinformation in the market (Table 6).

### 6.2. Other Countries and International Organizations

Research indicates that, internationally, there is a notable scarcity of standards governing selenium-enriched agriculture. Neither the WHO, CAC, nor major countries and regions worldwide have established definitive criteria for selenium-enriched agricultural products. As a result, no formal standards exist specifying selenium content in these agricultural commodities.

## 7. Standards for Selenium-Enriched Production Technical Procedures and Inputs

Agricultural products naturally containing selenium originate from soils with intrinsically high selenium concentrations, where inorganic selenium compounds undergo bioconversion into various organic selenium species. Conversely, artificially selenium-enriched agricultural products are generated through processes such as plant, animal, and microbial conversion. In these cases, supplemented inorganic selenium is deliberately transformed into different organic selenium forms to ensure adequate selenium intake [56].

Plant conversion is facilitated through agronomic practices, including inorganic soil fertilization, foliar feeding, and the application of selenium-enriched organic fertilizers. This method exploits plants’ innate ability to accumulate selenium, yielding selenium-enriched rice, fruits, vegetables, and other plant-derived foods [3]. Selenium-enriched animal products—such as meat, dairy, and eggs—are obtained by supplementing animal diets with selenium. Microbial conversion involves introducing inorganic selenium into culture media, where it interacts with proteins or polysaccharides to produce selenium-enriched yeasts and fungi [56,93]. Production technical guidelines provide comprehensive instructions and requirements for the cultivation and breeding of selenium-enriched agricultural products, ensuring that their selenium content meets prescribed standards. The potential environmental ramifications of selenium-enriched production techniques and selenium inputs cannot be underestimated; however, current research on this subject is limited. This area warrants increased attention in future studies to ensure sustainable practices and to mitigate any adverse environmental consequences.

The incorporation of selenium inputs represents a crucial technical strategy for producing artificially selenium-enriched agricultural commodities. By administering selenium-containing fertilizers or feeds, standardized products with uniform selenium content can be achieved [94]. During cultivation and breeding stages, many countries regulate selenium as an essential trace nutrient in animal nutrition, mandating its inclusion in animal feeds. However, except for Finland, no countries have established regulations for the supplementation of selenium in fertilizers.

### 7.1. China

Currently, China has promulgated over 60 local and group standards outlining technical guidelines to produce selenium-enriched products, yet national or industry-wide standards remain absent. These standards primarily address plant-based foods—such as rice and tea—as well as animal-derived products, like eggs and meat. They typically encompass sections on terminology and definitions, general requirements, cultivation techniques, harvesting protocols, packaging, transportation and storage procedures, and record management (Table 7).

The Regulations on the Safe Use of Feed Additives [95] define the maximum permissible dosages of selenium in animal feeds. Specifications mandate that sodium selenite should possess a purity of ≥98.0% (dry basis) when measured as the compound, or ≥44.7% (dry basis) when calculated as elemental selenium. For selenium-enriched yeast, the requirement is that the organic selenium content, expressed as elemental selenium, must be ≥0.1%. The recommended inclusion rate of selenium in compound feeds or total mixed rations for livestock, poultry, and aquaculture species—calculated as elemental selenium—is 0.1–0.3 mg/kg, with an upper limit of 0.5 mg/kg.

Selenium should first be formulated into a premix prior to use, and product labels must explicitly indicate the maximum selenium content. Labels are required to display the maximum selenium content, ensuring that the inorganic selenium fraction does not exceed the total selenium content. Currently, the standards regulating selenium inputs remain relatively underdeveloped.

**Table 7:** Some standards for selenium-enriched products production technical regulations of China *.

Standard Number	Standard Name	Region
DB36/T 1112-2019 [96]	Technical Regulations for Selenium-enriched Rice Production	Jiangxi
DB36/T 1322-2020 [97]	Technical regulations for the production of selenium-enriched eggs	Jiangxi
DB45/T 2554-2022 [98]	Technical regulations for the production of selenium-enriched shiitake mushrooms	Jiangxi
DB61/T 307.2-2021 [99]	Ziyang selenium-enriched tea production technical regulations	Shaanxi
DB61/T 557.4-2012 [100]	Technical regulations for production of selenium-enriched pork pigs	Shaanxi
T/FXXH 010-2020 [101]	Technical regulations for production of selenium-enriched wheat	Zibo Selenium-Enriched Agricultural Products Association
T/KYFX 5-2019 [102]	Kaiyang Ecological Selenium-enriched Egg Production Technical Regulations	Kaiyang Selenium-Enriched Products Association
T/HBSE 0004-2019 [103]	Technical Regulations for the Production of Selenium-Enriched Rice	Hubei Province Selenium Industry Association
T/HBSE 0005-2019 [104]	Technical Regulations for the Production of Shien Selenium-Enriched Tea	Hubei Province Selenium Industry Association
T/CI 080-2023 [105]	Technical Regulations for the Production of Organic Selenium-Enriched Navel Orange	China International Association for Promotion of Science and Technology

* Local standards and group standards only list part of the standards.

### 7.2. Other Countries and International Organizations

Developed nations and international organizations have established relatively few technical guidelines for the production of selenium-enriched products. However, certain selenium-deficient developed countries have implemented specific regulations and standards to manage selenium-containing inputs in agricultural and livestock sectors.

Since 1985, nearly all compound fertilizers in Finland have been supplemented with selenium. The Decree of the Ministry of Agriculture and Forestry on Fertiliser Products [106] stipulates that, in solid fertilizers, selenium may be added in the form of selenate at concentrations of 15–20 mg/kg (dry weight). In liquid fertilizers, selenium can also be added as selenate, with a maximum concentration of 15 mg/kg. When applying liquid selenium-containing fertilizers to soil, the application rate must not exceed 10 g/ha. For foliar applications, the selenium addition must not exceed 4 g/ha per growth cycle.

In Australia and New Zealand, selenium used as a fertilizer ingredient is regulated under the Biosecurity Act 2015 [107] and the Australia New Zealand Food Standards Code [108]. Australia’s Agricultural and Veterinary Products (Control of Use) Regulations 2017 [109] mandate that, if the selenium content in fertilizers exceeds 10 mg/kg, a warning label must be included, indicating that excessive use may be toxic to livestock. Specifically, for pasture applications, overuse is prohibited unless a selenium deficiency is present. In New Zealand, the recommended selenium content for animal feed is 0.03 mg/kg of dry matter.

The Canadian Food Inspection Agency, through its Safety Standards for Fertilizers and Supplements (T-4-93) [110], requires that the acceptable maximum selenium concentration in products be determined based on the maximum allowable cumulative addition levels in soil.

In the United States, the maximum allowable selenium concentration in animal feed is set at 0.3 ppm per ton of complete feed, applicable to livestock, such as chickens, turkeys, pigs, and cattle. For cattle supplements, selenium intake must not exceed 3 μg per cow per day. In free-choice salt–mineral mixtures for cattle, the selenium content is limited to 120 ppm per ton, with a daily intake not exceeding 3 μg.

The European Union has authorized selenium-enriched yeast for use in animal feeds. According to Regulation (EC) No. 1831/2003 [111], the maximum supplementary selenium level is established at 0.2 mg/kg of complete feed with a moisture content of 12%.

Despite these regulations, current technical guidelines for selenium-enriched agriculture are incomplete, and there is insufficient evidence for comprehensive risk assessment. Many countries have not clearly defined the types of selenium sources permissible for addition to fertilizers, their quality specifications, or appropriate usage and dosages. There is also a lack of technical guidelines for the scientific application of fertilizers based on classification. Moreover, methods for detecting organic selenium are expensive and require further optimization. No mandatory control standards or regulations are in place to address risks associated with improper selenium application during agricultural cultivation, such as the direct foliar spraying of inorganic selenium onto crops. The potential risks that selenium-enriched production poses to the environment, human populations, and flora and fauna urgently need thorough assessment.

## 8. Standards for Selenium-Enriched Certification

The implementation of selenium-enriched food certification plays a pivotal role in safeguarding consumer rights, guiding consumption behaviors, and further enhancing the quality of selenium-enriched food products. Moreover, it promotes the healthy and sustainable development of the selenium-enriched food industry.

### 8.1. China

Regarding certification standards, the Certification and Accreditation Administration of China (CNCA) has issued RB/T138-2023 [112], Technical Specifications for Selenium-Enriched Product Certification. This standard applies to certification activities for selenium-enriched products conducted by third-party certification bodies and may also serve as a reference for organizations producing such products and related institutions. It provides detailed product classifications and specifies general requirements for certification, including evaluation criteria, implementation procedures, and guidelines for certification labeling. The standard is intended to guide third-party certification bodies in the certification process of selenium-enriched products, as well as to assist production organizations and other pertinent institutions.

**Figure 4:**
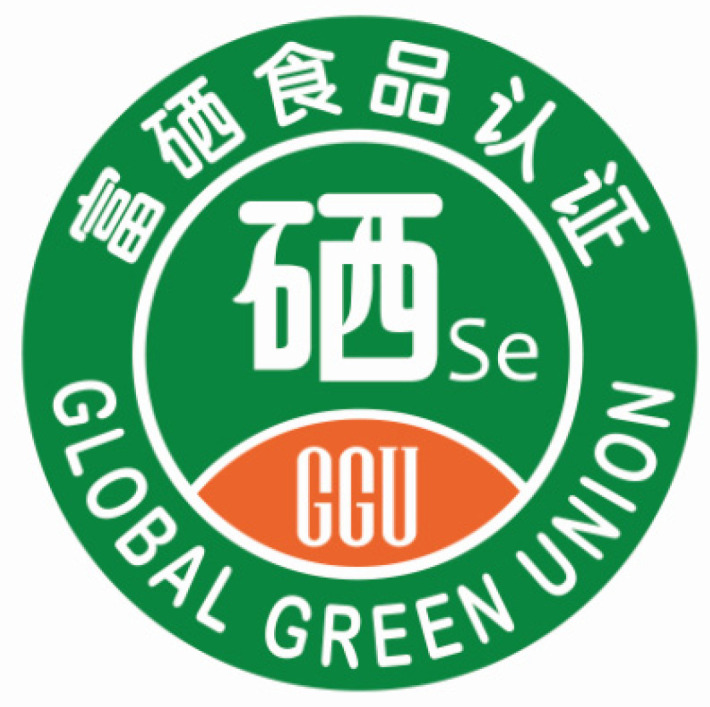
Selenium-enriched food certification mark of the Global Green Alliance. Foods reviewed by the Global Green Alliance for Selenium Enrichment requirements can receive this mark to certify as selenium-rich foods.

In addition to national standards, certain selenium-enriched regions and organizations have obtained certification qualifications or established their own certification rules based on these guidelines. For instance, the Ankang Tea Association in Shaanxi Province introduced the Implementation Rules for the Use of the Selenium-Enriched Tea Geographical Indication Certification Trademark. Similarly, Enshi Prefecture in Hubei Province established its own “organic selenium-enriched” certification system. The Global Green Alliance (Beijing) Food Safety Certification Center developed a selenium-enriched food certification system grounded in the Regulations of the People’s Republic of China on Certification and Accreditation, the Food Safety Law of the People’s Republic of China, and the Dietary Guidelines for Chinese Residents. This system sets forth regulations for selenium-enriched food certification, including the certification subjects, basic application requirements, and scope of certification. Only products that successfully pass the selenium-enriched food certification are permitted to use the certification mark on their packaging, labels, advertisements, promotional materials, and instruction manuals, indicating that they have met the established certification requirements (Figure 4).

### 8.2. Other Countries and International Organizations

Currently, developed countries such as the United States and Japan have implemented regulations concerning selenium-fortified foods, particularly providing clear guidelines on food safety, nutritional fortification, and labeling requirements. In these nations, the certification of selenium-enriched products is often manifested through nutritional claims or labels rather than through a dedicated “selenium-enriched food” certification process. For example, within the European Union, the certification and management of selenium-enriched foods—or any novel foods—is primarily governed by the EU Novel Food Regulation (Regulation (EU) 2015/2283) [80]. According to this regulation, novel foods are defined as those not widely consumed within the EU before 15 May 1997, which includes selenium-enriched foods. If selenium-enriched foods are produced using new technologies or contain novel ingredients, they may be classified as novel foods under this regulation.

## 9. Summary and Outlook

To address challenges, such as inconsistent quality and an incomplete standardization system in selenium-enriched agriculture, the following recommendations aim to enhance standard quality, advance the selenium-enrichment industry, and regulate the market effectively.

First, it is imperative to strengthen the safety risk assessment of selenium-enriched agricultural products. Regions abundant in selenium resources often coincide with areas of heavy metal accumulation, leading to selenium-enriched products frequently exceeding permissible heavy metal limits [113,114]. Current research predominantly focuses on the accumulation and residue levels of selenium inputs within plants and animals themselves [115]. It is recommended to conduct comprehensive studies on the metabolism and accumulation patterns of selenium during the cultivation of key agricultural commodities, while continuously monitoring selenium nutritional status across diverse regions and populations. Attention should also be directed towards the impact of selenium-enriched agriculture on soil and water quality, avoiding excessive fertilizer use that could exacerbate environmental issues. A systematic evaluation of safe threshold levels for selenium content in agricultural products should be undertaken to ensure human health and provide a foundation for developing selenium-enriched agriculture standards.

Second, establishing and refining the standard system for selenium-enriched agriculture is crucial. This involves researching and accelerating the formulation of standards for defining selenium-enriched agricultural products and their labeling requirements. Implementing clear regulations for production, oversight, and consumer information of selenium-enriched products not only fortifies their scientific foundation but also enhances production traceability. This ensures that consumers can readily identify the selenium-enriched attributes and nutritional value of these products, thereby increasing market transparency. There is a need to investigate the criteria for adding selenium to feeds and fertilizers and develop cultivation and input standards specific to selenium-enriched agricultural products within the agricultural sector. Ensuring the scientific and judicious application of selenium in agricultural production fortifies the rigor and practicality of farming practices while guaranteeing consistent product quality. Proper selenium management mitigates environmental issues and product quality instability that may arise from both excessive and insufficient use. Additionally, standards for organic selenium detection methods should be developed to align with practical agricultural needs, to ensure the safety and stability of selenium-rich products.

Third, enhancing quality and safety supervision mechanisms is essential, to improve the applicability of existing standards, enforce relevant regulations, and elevate both regulatory efficiency and product quality, along with the development of a robust selenium-enriched certification system and promotion of the widespread adoption of labeling standards. Efforts should also be intensified to combat counterfeit and substandard products to maintain market integrity. Augmenting the quality and safety regulations for selenium-enriched products, while ensuring the applicability and practicality of existing standards, can substantially enhance regulatory efficiency across the entire production and distribution continuum. This approach not only elevates product quality but also forestalls the entry of substandard or non-compliant products into the market. A stringent regulatory framework ensures that products comply with standards throughout the entire supply chain—from production to retail—thereby promoting market stability. Furthermore, the establishment of a certification system can amplify consumer trust, deter counterfeit products, and propel the sustainable and robust development of the industry.

## Figures and Tables

**Table 1:** Recommended Intake (RNI) and Tolerable Upper Intake Levels (UL) for selenium (μg/day).

Age		WHO		DE		Nordic Europe		EU		AU/NZ		US		UK		JP		CHN	
		RNI *	UL *	RNI	UL	RNI	UL	AI *	UL	RNI	UL	RNI	UL	RNI	UL	RNI	UL	RNI	UL
Infant (Months)	0–3	6		10						12	45	15	45	10				15	55
	4–6	6		15						12	45	15		13				15	55
	7–12	10		15		15		15	55	15	60	20	60	10				20	80
Children	1–2	17	90	15		20		15	70	25	90	20	90	15		10	100	25	80
	2–4	17	90	15		25		15	70	25	90	20	90	15		15	100	25	120
	4–8	21	150	20		30		20	95	30	150	30	150	20		15	100	40	150
	8–10	21	280	30		30		35	130	50	280	40	280	30		20	120	45	200
	10–13	34	280	43		40		55	180	50	280	40	280	45		25	160	60	300
	13–15	34		60		60		55	180	70	400	55	400	45		30	210	60	300
Male	15–18	34		70				70	230	70	400	55	400	45		35	260	60	350
	18–70	34	400	70	400	60	300	70	255	70	400	55	400	75	450	30	300	60	400
	>70	34		70	400	60	300	70	255	70	400	55	400	75	450	30	260	60	400
Female	15–18	26		60		50		70	255	60	400	55	400	60		25	220	60	350
	18–70	26	400	60	400	50	300	70	255	60	400	55	400	60	450	25	230	60	400
	>70	26		60	400	50	300	70	255	60	400	55	400	60	450	25	210	60	400
Pregnant Women		28		60		60		70	255	65	400	60	400			30		65	400
Lactating Women		35		75		60		85	255	75	400	70	400	75		45		78	400

* RNI: Recommended Nutrient Intake. UL: Tolerable Upper Intake Level. AI: Adequate Intake.

**Table 2:** Selenium content testing standards of China.

Standard Name	Standard Number	Standard Category	Applicable Subjects	Main Content
National Food Safety Standard Determination of Selenium in Food	GB 5009.93-2017 [42]	National Standards	Food	Specified the determination of total selenium by AFS, FSP, and ICP–MS.
National Food Safety Standard Food Nutrition Fortifier Selenium-enriched Edible Fungus Powder	GB 1903.22-2016 [43]	National Standards	Edible fungus powder	Specified methods for the determination of total selenium, inorganic selenium, and organic selenium.
Inspection of grain and oils—Determination of sodium, magnesium, kalium, calcium, chromium, manganese, iron, copper, zinc, arsenic, selenium, cadmium and plumbum in cereals and derived products—Inductively coupled plasma-mass spectrometric method	GB/T 35876-2018 [44]	National Standards	Cereals and derived products	Specified the determination of total selenium by ICP–MS.
Determination of seleno-cystine, methyl-nitro-cysteine and seleno-methionine in livestock and poultry meat by high performance liquid chromatography–atomic fluorescence spectrometry	NY/T 3947-2021 [45]	Agricultural industry standard	Livestock and poultry meat	Specified the methods for the determination of organic selenium forms in poultry and livestock meat.
Determination of seleno-amino acids in seleno-proteins by liquid chromatography–atomic fluorescence spectrometry [45]	NY/T 3870-2021 [46]	Agricultural industry standard	Seleno-proteins	Specified methods for the determination of selenium-containing amino acids.
Determination of selenocysteine and seleno-methionine in grains by liquid chromatography–inductively coupled plasma mass spectrometry	NY/T 3556-2020 [47]	Agricultural industry standard	Grains	Specified the methods for the determination of organic selenium forms in grains.
Determination method of selenium in exported foods	SN/T 0860-2016 [48]	Import and export industry standards	Exported foods	Specified methods for the determination of total selenium.

**Table 3:** Selenium content testing standards of other countries and international organizations.

Country/ Organization	Standard Name	Standard Number	Applicable Subjects	Main Content
ISO	Water Quality—Determination of Selenium—Part 1: Method using Hydride Generation Atomic Fluorescence Spectrometry (HG–AFS)	ISO/TS 17379-1:2013 [49]	Drinking water, surface water, groundwater, and rainwater	Specified the methods for the determination of selenium. The applicable range is 0.02 μg/L to 100 μg/L. This method is unlikely to detect organic selenium compounds.
	Water Quality—Determination of Selenium—Part 2: Method using Hydride Generation Atomic Absorption Spectrometry (HG–AAS)	ISO/TS 17379-2:2013 [50]		
	Water Quality—Application of Inductively Coupled Plasma Mass Spectrometry (ICP–MS)—Part 2: Determination of Selected Elements, Including Uranium Isotopes	ISO 17294-2:2023 [51]	Drinking water, surface water, groundwater, wastewater, and leachate	Specified the determination of selenium, silver, and other metallic elements, as well as uranium and its isotopes, in water.
	Infant Formula and Adult Nutritional Products—Determination of Chromium, Selenium, and Molybdenum—Inductively Coupled Plasma Mass Spectrometry (ICP–MS)	ISO 20649:2015 [52]	Infant formula and adult nutritional formula	Specified the quantitative determination methods for chromium, selenium, and molybdenum.
EU	Hydride Generation Atomic Absorption Spectrometry (HG–AAS)	EN 14627:2005 [53]	Food	This method quantifies selenium concentration by detecting selenium atoms in the gas phase.
USA	AOAC Official Method 2011.19		Infant formulas and adult nutritional products [54]	Using Inductively Coupled Plasma Mass Spectrometry, it is primarily used for the detection of selenium, chromium, and molybdenum.
	AOAC 984.27		Food [55]	Selenium in food is detected using Hydride Generation Atomic Absorption Spectrometry, suitable for low-concentration selenium samples.
	AOAC 996.16		Food [56]	Selenium compounds in food are determined using Inductively Coupled Plasma Optical Emission Spectrometry, suitable for complex food samples.

**Table 4:** Standards for selenium-enriched foods in China.

Standard Name	Standard Number	Standard Category	Applicable Subjects	Main Content
National Food Safety Standard Food Nutritional Fortifier Seleno-protein	GB 1903.28-2018 [66]	National Standard	Seleno-protein	1000–2500 mg/kg seleno-methionine, selenium-enriched, with organic selenium accounting for ≥80% of the total selenium by mass.
National Food Safety Standard Food Nutrition Fortifier Selenium-enriched Yeast	GB 1903.21-2016 [67]	National Standard	Yeast	1000–2500 mg/kg, selenium-enriched; with organic selenium accounting for ≥97% of the total selenium by mass.
National Food Safety Standard Food Nutrition Fortifier Selenium-enriched Edible Fungus Powder	GB 1903.22-2016 [68]	National Standard	Edible Fungus Powder	180–400 mg/kg, selenium-enriched; with organic selenium accounting for ≥98% of the total selenium by mass. Specified methods for the determination of total selenium, inorganic selenium, and organic selenium.
National Food Safety Standard Food Nutritional Enhancer Selenized Carrageenan	GB 1903.23-2016 [69]	National Standard	Carrageenan	2%, selenium-enriched, with organic selenium accounting for ≥90% of the total selenium by mass.
National Food Safety Standard Food Nutrition Fortifier Sodium Selenite	GB 1903.9-2015 [70]	National Standard	Sodium Selenite	100.8% ≥ Sodium Selenite ≥ 96.4%
National Food Safety Standard Food Nutrition Fortifier L-Selenium-Methyl-selenocysteine	GB 1903.12-2015 [71]	National Standard	L-Selenium-Methyl-selenocysteine	L-Selenium-Methyl-selenocysteine ≥ 96%
National Food Safety Standard For the Usage of Nutrition Enrichment	GB 14880-2012 [65]	National Standard	Nutrition Enrichment	The sources of selenium nutritional fortifiers and the permitted range of selenium addition in food have been specified.
National Food Safety Standard General Rules for Nutrition Labeling of Prepackaged Foods	GB 28050-2011 [64]	National Standard	Prepackaged Foods	The nutritional claims for “selenium-enriched” and “high-selenium” have been specified: Selenium ≥ 18 µg/100 g.
Fundamental Terminology and Definition of Nutritional Component in Foods	GB/Z 21922-2008 [72]	National Standard	Nutritional Component in Foods	Selenium is classified as an essential inorganic chemical element necessary for maintaining normal physiological functions in the human body.
Reference Intake of Dietary Nutrients for Chinese Residents Part 3: Trace Elements	WS/T 578.3-2017 [73]	Health Industry Standard	Chinese Residents	The recommended intake and tolerable upper intake levels of selenium have been specified.

**Table 5:** Standards for selenium-enriched foods of other countries and international organizations.

Country/Region	Standard Name	Main Content
CAC	Codex Guidelines for Use of Nutrition and Health Claims (CAC/GL 23-1997) [74]	If the nutrient content reaches 30% of the NRV, it can be claimed as “high content” ≥16.5 µg/100 g.
EU	90/496/EEC [74] (EU) No 1924/2006 [76]	If the nutrient content reaches 30% of the NRV, it can be claimed as “high content” ≥16.5 µg/100 g.
USA	CFR—Code of Federal Regulations Title 21 [77]	If the nutrient content reaches 20% of the NRV, it can be claimed as “rich in” ≥11 µg/100 g
AU/NZ	Australia New Zealand Food Standards Code [78]	17.5 µg can be labeled as selenium-enriched food.

**Table 6:** Some standards for selenium-enriched agricultural products of China *.

Standard Name	Standard Number	Standard Category	Applicable Subjects	Main Content
Rich-selenium paddy	GB/T 22499-2008 [82]	National Standards	Rich	0.04–0.30 mg/kg, selenium-enriched; >0.30 mg/kg, selenium exceeds the limit.
Selenium-enriched potatoes	NY/T 3116-2017 [83]	Agricultural Industry Standards	Potatoes	15–150 μg/kg, selenium-enriched.
Selenium-enriched garlic	NY/T 3115-2017 [84]	Agricultural Industry Standards	Garlic	30–300 μg/kg, selenium-enriched.
Rich-selenium tea	NY/T 600-2002 [85]	Agricultural Industry Standards	Tea	0.25–4.00 μg/kg, selenium-enriched.
Selenium-rich potatoes	GH/T 1310-2020 [86]	China Supply and Marketing Cooperation Industry Standard	Potatoes	0.015–0.150 mg/kg
Selenium-rich agricultural products	GH/T 1135-2017 [87]	China Supply and Marketing Cooperation Industry Standard	Agricultural Products	The upper and lower limits of selenium content in various agricultural products are stipulated.
Rich-selenium tea	GH/T 1090-2014 [88]	China Supply and Marketing Cooperation Industry Standard	Tea	0.2–4.0 mg/kg, Selenium-enriched
Food Safety Local Standards Requirements for Selenium Content in Organic Selenium-rich Foods	DBS42/002-2021 [89]	Hubei Provincial Food Safety Local Standard	Cereals, vegetables, fruits, edible fungi, tea, livestock and poultry products and their products, etc.	Cereals: 20.0–50.0 μg/100 g Potatoes: 20.0–100.0 μg/100 g Garlic: 20.0–200.0 μg/100 g Tea: 20.0–500.0 μg/100 g
Selenium Content Requirements for Selenium-enriched Agricultural Products	DB13/T 2702-2018 [90]	Hebei Provincial Standard	Cereals, vegetables (fresh weight), fruits and products, potatoes (fresh weight)	Cereals: 0.10–0.80 mg/kg Potatoes (fresh weight): 0.02–0.50 mg/kg
The Grain & Oil Products of Shaanxi-Ankang Rich-selenium rice	T/SAGS 013-2021 [91]	Shaanxi Grain Industry Association	Rice	0.15–0.6 mg/kg, Selenium-enriched
Selenium-enriched pomegranate	T/FXXH 016-2020 [92]	Zibo Selenium-rich Agricultural Products Association	Pomegranate	0.10–0.15 mg/kg, Selenium-enriched

* Local standards and group standards only list part of the standards.

## Abbreviations

WHO: World Health Organization; RNI, recommended intake; UL, tolerable upper intake levels; NOAEL, no observed adverse effect level; LOAEL, lowest observed adverse effect level; UF, uncertainty factor; EFSA, European Food Safety Authority; NDA, Novel Foods and Food Allergens; AI, adequate intake; EAR, estimated average requirement; RDA, recommended dietary allowance; LD_50_, lethal dose; LC–ICP–MS, liquid chromatography–inductively coupled plasma mass spectrometry; ICP–MS, inductively coupled plasma mass spectrometry; ISO, International Organization for Standardization; SAMR, State Administration for Market Regulation; NRV, Nutrient Reference Value; CAC, Codex Alimentarius Commission; EU, European Union; FDA, Food and Drug Administration; DSHEA, Dietary Supplement Health and Education Act; CGMP, Current Good Manufacturing Practices; CNCA, Certification and Accreditation Administration of China.
